# Long-term care insurance and labor-force participation of adult children: an analysis of substitution and anticipation effects

**DOI:** 10.3389/fpubh.2025.1601077

**Published:** 2025-05-14

**Authors:** Yang Yi, Jinghong Gu, Qinglin Xu, Hai Gu

**Affiliations:** ^1^Center for Health Policy and Management Studies, School of Government, Nanjing University, Nanjing, China; ^2^Department of Health Policy and Management, Columbia University Mailman School of Public Health, New York, NY, United States

**Keywords:** labor-force participation, long term care insurance, substitution effects, anticipation effects, China

## Abstract

**Background:**

Many informal caregivers at working age and face the dual burden of providing care and working. This study examines how China’s long-term care insurance (LTCI) pilot programs affect the labor-force participation of adult children who may provide informal care to parents.

**Methods:**

We analyze four waves (2011, 2013, 2015, and 2018) of micro panel data from the China Health and Retirement Longitudinal Study and exploit the staggered rollout of LTCI pilots across cities from 2012 to 2017. A difference-in-differences design estimates the causal impact of LTCI implementation on labor-force participation of adult children, with robustness checks and subgroup analyses by gender, age, cohabitation status, and skill level.

**Results:**

Implementation of LTCI significantly increases the likelihood of adult children remaining in the labor force. Mechanism analysis indicates this effect is driven by both reduced caregiving time (substitution effect) and improved expectations of future support (anticipation effect). The positive impact is particularly strong among men, individuals under 45 years old, cohabitation without parents, and lower-skilled workers.

**Conclusion:**

Expanding LTCI can effectively alleviate the caregiving-employment conflict and enhance labor participation of adult children. To maximize workforce and social welfare benefits, policymakers should expand LTCI coverage, strengthen community care services, and focus support on high-burden caregiver groups.

## 1 Introduction

The organization and provision of long-term care (LTC) is a key challenge in aging societies, as the growing demand for LTC services necessitates effective policy responses (1, 2). Among countries of the Organization for Economic Cooperation and Development, many have introduced insurance schemes to increase the supply of LTC services (3, 4). While policymakers typically focus on the direct effects of long-term care insurance (LTCI) on the health outcomes of older adults, its spillover effects on family members—particularly in terms of labor supply—also warrant attention (5–7).

In general, LTC needs can be met by both formal and informal care (8, 9). Formal care involves paid services provided by professional institutions (e.g., hospitals, nursing facilities) and personnel (e.g., nurses, caregivers), whereas informal care refers to unpaid services delivered by family members and friends (10–12). Prior research has shown that informal care often competes with paid employment, which can reduce caregivers’ labor participation (13). Accordingly, LTCI may help alleviate caregivers’ burdens by expanding access to affordable formal care resources, potentially “substitution” informal care while supporting increased labor force participation among family caregivers (9, 14, 15). When parents hold LTCI coverage, a portion of their future care costs is covered, thereby reducing the financial and temporal caregiving burden on their adult children (16).

Thus, LTCI influences informal caregivers’ trade-off between labor supply and care provision. On one hand, meeting LTC needs can divert time away from employment (17). On the other hand, increased caregiving expenditures may motivate higher labor force participation. Some scholars have analyzed the implementation of LTCI policies from the perspective of liberating and returning family labor to the labor market. Prior to the implementation of LTCI policies, the care of the older adults in China, especially the disabled older adults, was dominated by informal family caregiving, which inhibited adult children’s self-employment (13). Informal caregiving responsibilities have a significant negative impact on the labor supply and life satisfaction of family caregivers (18, 19). In contrast, after the implementation of LTCI, caregiving institutions continue to grow, and family members anticipate that disabled older adults who are eligible for caregiving and allowed by their financial circumstances will be able to receive professional caregiving services, which will greatly reduce the caregiving pressure on family members (20), thus family workforce can return to the labor market and significantly increase labor force participation.

Existing studies have examined the relationship between LTCI and labor-force participation of adult children (6, 21, 22), yet few have accounted for the insurance’s intended effects on household members who do not yet have incapacitated parents. In contexts where parents are already disabled or demented, LTCI’s substitution effect—the provision of formal care that alleviates adult children’s caregiving burden and enables them to restore or increase labor-force participation—may yield only modest supply gains. By contrast, LTCI’s anticipation effect—the expectation of coverage that reduces uncertainty about future care needs and thereby strengthens adult children’s work motivation and labor-supply decisions—could produce more substantial employment benefits, even for households whose parents remain healthy.

This paper uses a difference-in-differences (DID) design and a series of robustness checks to evaluate the impact of China’s LTCI implementation on labor-force participation of adult children. Our research contributes in the following aspects. First, by explicitly considering the uncertainty surrounding parental incapacitation and dementia, we highlight LTCI’s role in mitigating this uncertainty and motivating adult children’s employment decisions. Second, building on the classic substitution effect, we further investigate how expectations about future care burdens—what we term the “expectation effect”—mediate LTCI’s impact on employment decisions. By disentangling substitution from expectation pathways, we shed light on the behavioral underpinnings of LTCI-induced labor-supply changes (21, 23–25). Third, we extend prior research by examining differences in employment outcomes across skill levels, shedding light on how LTCI may influence high-, medium-, and low-skilled labor-force participation of adult children. Finally, our findings have practical policy relevance for optimizing and expanding LTCI programs.

## 2 Materials and methods

### 2.1 The LTCI program

LTCI is a form of health insurance that compensates for the costs of care services required by individuals due to old age, illness, or disability. Originating in the United States in the 1970s, LTCI later expanded to European countries such as France, Germany, the United Kingdom, Ireland, and South Africa. In 2000, Japan formally integrated LTC coverage as a public service product into its national social security system. Although LTCI has achieved considerable global uptake, it remains in its early stages in China.

In 2016, the Ministry of Human Resources and Social Security issued *the Guiding Opinions on Carrying Out the Pilot LTCI System*, which outlined fundamental policies, administrative guidelines, and supportive measures for LTCI. Fifteen cities—including Chengde, Changchun, and Qiqihar—were designated as pilot sites. Under this policy, LTCI covers participants of basic medical insurance, with differentiated coverage levels and payment rates according to care needs and service modalities and reimburses a proportion of incurred care costs.

In 2020, the National Healthcare Security Administration, in collaboration with the Ministry of Finance, introduced *the Guiding Opinions on the Expansion of the LTCI System Pilot Program*, advocating the development of a more comprehensive LTCI framework. By the end of 2023, China’s LTCI pilot program had expanded to 49 regions, covering 183.31 million people, including 1.34 million who were already receiving LTCI benefits. The pilot involved 8,080 designated service organizations and 302,800 nursing service personnel, reflecting the rapid advancement of LTCI in China.^1^

### 2.2 Econometric specification

To estimate the effect of LTCI policy implementation on labor-force participation of adult children, we exploit LTCI pilots reforms during the sample period as a quasi-natural experiment. Specifically, we employ a DID approach to compare changes in labor-force participation in newly implemented LTCI regions versus non-LTCI regions, before and after the policy’s introduction. Given our use of panel data and binary explanatory variables, we specify the baseline model as an xtprobit regression (see Equation 1):


(1) $$y_{\mathrm{ikt}}=\alpha_{0}+\alpha_{1}\mathrm{pos}t_{\mathrm{ikt}}\cdot \text{trea}t_{\mathrm{ikt}}+\sum_{j=1}^{m}\alpha_{j}X_{\mathrm{ikt}}^{j}+\lambda_{t}+\gamma_{k}+\lambda_{t}\cdot \gamma_{k}+\varepsilon_{\mathrm{ikt}}$$

Among them, $\text{trea}t_{\mathrm{ikt}}$ is the LTCI pilot dummy variable, which was set to 1 if in a LTCI pilot region and eligible for the type of health insurance coverage, and 0 otherwise; $\mathrm{pos}t_{\mathrm{ikt}}$ is the LTCI pilot period dummy variable, which was set to 1 for the period when LTCI was carried out and for each period thereafter, and 0 otherwise; $\mathrm{pos}t_{\mathrm{ikt}}\cdot \text{trea}t_{\mathrm{ikt}}$ is the independent variable, a dummy variable for the implementation of LTCI, which is 1 for the period when LTCI was implemented in the individual’s region and each subsequent period, and 0 otherwise. If its coefficient $\alpha_{1}$ is less than 0, LTCI reduced the likelihood of offspring’s labor force participation. If it is greater than 0, LTCI raised the likelihood of offspring’s labor force participation. $X_{\mathrm{ijt}}$ is an individual- and household-level control variable, with $\alpha_{j}$ is its estimated coefficient; $\alpha_{0}$ is a constant term, $\lambda_{t}$ is a time fixed effect, $\gamma_{k}$ is an city fixed effect, $\lambda_{t}\cdot \gamma_{k}$ is the cross-multiplication term of the time fixed effect and the city fixed effect, and $\varepsilon_{\mathrm{ikt}}$ is a randomized disturbance term.

### 2.3 Data

We draw on individual-level data from the 2011, 2013, 2015, and 2018 waves of the China Health and Retirement Longitudinal Study (CHARLS). CHARLS samples Chinese residents aged 45 and above (and their spouses) across approximately 10,000 households in 150 cities, totaling around 17,000 respondents. In addition to collecting standard demographic and socioeconomic information, the survey gathers extensive details on health status, health service utilization, health insurance, income and consumption, financial support, and caregiving. Notably, CHARLS includes questions on each household’s children, including their employment status, thereby enabling this study’s focus on labor-force participation of adult childrenlabor-force.

In defining the sample, adult children were restricted to those aged 16 to 60. This criterion aligns with China’s labor regulations, which prohibit the employment of minors under 16 (*Labor Law of the People’s Republic of China*) and typically recognize 60 as the retirement age. Observations missing key variables, including age, gender, work status, and income, were excluded. After constructing an unbalanced panel, the final analytic sample contains 38,847 valid person-period observations, with adult children as the primary unit of analysis. Information on LTCI coverage was collected from official policy documents of pilot cities. China has introduced LTCI in phases, beginning with *Qingdao’s Opinions on Establishing a Long-Term Medical Care Insurance System (Trial)* in July 2012, followed by national guidance in 2016 and again in 2020. Given data constraints and the fact that the second round of national pilot sites was launched in 2020, this study focuses on the first set of pilot regions. Two pilot sites—Nantong City in Jiangsu Province and Shihezi City in the Xinjiang Production and Construction Corps—were excluded due to unavailability of relevant data. Ultimately, 13 pilot cities (e.g., Chengde City in Hebei Province and Qingdao City in Shandong Province) included in CHARLS are classified as the treatment group, while all remaining cities serve as the control group.

### 2.4 Variables

#### 2.4.1 Dependent variable

The principal dependent variable is labor-force participation among adult children. In CHARLS, each child respondent was asked, “Are you currently working or attending school?” Those who answered “working” were coded as 1, and those who selected any other response were coded as 0.

#### 2.4.2 Independent variable

The key independent variable is a dummy indicator representing LTCI pilot status. It equals 1 if an individual resides in a city that implemented LTCI during or after a specified pilot start date, and 0 otherwise.

#### 2.4.3 Control variable

Control variables encompass characteristics at both the child- and parent-level. At the child level, we control age, gender, marital status, household registration (hukou) status, and education. At the parent level, we include age, age-squared, gender, marital status, household registration status, education level, self-assessed health, smoking, alcohol consumption, chronic disease count, medical insurance, and disability status.

Among them, age is calculated as the difference between the interview year and birth year. An age-squared term captures potential nonlinear effects; gender is a binary variable, with 1 for males and 0 for females; marital status is a binary variable, with 1 for being in a marriage and 0 otherwise; household registration status is a binary variable, with 0 for rural and 1 for non-rural; education level is an ordered categorical variable (0–5), indicating below elementary school, elementary school, middle school, high school, bachelor’s degree (specialist), graduate school and above; self-assessed health is an ordered categorical variable (1–5), indicating very bad, bad, fair, good, and very good health levels; smoking and alcohol consumption are both binary variables, with smokes or consumes alcohol as 1 and 0 otherwise; chronic disease count is a continuous variable ranging from 0 to 10, reflecting the number of chronic conditions diagnosed; the medical insurance is a binary variable distinguishing between coverage for urban workers or urban/rural residents; disability is a dichotomous variable, according to Mahoney and Barthel’s (26) ability to perform daily living scale, respondents with a score above 60 are classified as having a disability 1, and 0 otherwise. All regressions incorporate time fixed effects to account for period-specific shocks and city fixed effects to control unobserved heterogeneity across regions. These specifications help isolate the causal impact of LTCI on labor-force participation of adult children labor-force.

### 2.5 Descriptive statistics

In order to align our sample with the study’s focus on individuals who may require long-term care, we first restrict the parent sample to those aged 60 or older—consistent with the standard LTCI coverage criteria for older adults with disabilities. Second, we remove outliers on key variables, as well as observations with missing essential data. This results in a final pooled sample of 38,800 person-wave observations, of which 3,270 are classified as covered by LTCI (i.e., they reside in a pilot region and are in a period after the pilot’s introduction). We further divide the dataset into LTCI-covered and LTCI-uncovered subsamples and compare mean differences.

Table 1 presents the descriptive statistics for the total sample and by LTCI coverage. Overall, 86.65% of adult children in the sample report being employed. In the LTCI-covered subsample, the labor-force participation rate is slightly higher than in uncovered regions, providing an initial indication that LTCI may positively influence labor-force participation of adult children labor-force. The remaining variables capture individual and parental characteristics (e.g., gender, age, health status), which serve as controls in our subsequent econometric analyses.

**Table 1:** Descriptive statistics.

Variables	Main sample					Treat group	Control group
	Obs	Mean	Std. dev.	Min	Max	Mean	Mean
*Cwork*	38,847	0.8665	0.3401	0	1	0.8697	0.8662
*Cgender*	38,847	0.5069	0.5	0	1	0.5144	0.5062
*Cage*	38,847	42.5891	7.4848	16	60	42.9514	42.5558
*Ceducation*	38,847	1.6118	1.1951	0	5	1.5869	1.6141
*Cmarry*	38,847	0.9458	0.2264	0	1	0.9424	0.9461
*Chukou*	38,500	0.2408	0.4276	0	1	0.2465	0.2403
*Cchildnum*	38,847	1.7602	0.9545	0	10	1.5113	1.7831
*Ctongzhu*	38,847	0.0797	0.2709	0	1	0.082	0.0795
*Clnin*	38,200	7.1838	3.4501	0	15.1892	7.2233	7.1801
*Cbro*	38,847	3.2507	1.7681	0	15	3.0471	3.2694
*Pgender*	38,847	0.5051	0.5	0	1	0.4951	0.506
*Page*	38,847	71.0072	7.362	60	103	70.6459	71.0404
*Page2*	38,847	5096.221	1074.961	3,600	10,600	5045.122	5100.918
*Phukou*	38,847	0.1917	0.3936	0	1	0.1895	0.1919
*Peducation*	38,847	0.8983	0.8912	0	4	0.9369	0.8948
*Pmarry*	38,847	0.5976	0.4904	0	1	0.6419	0.5935
*Psubjecthealh*	38,847	3.1002	0.7571	1	5	3.0697	3.103
*Psmoke*	38,847	0.1908	0.3929	0	1	0.1951	0.1904
*Pdrink*	38,847	0.3007	0.4586	0	1	0.3229	0.2987
*Pchronic*	38,847	0.6655	1.1939	0	10	0.6642	0.6656
*Pmedical*	38,847	0.3142	0.4642	0	1	0.2792	0.3175
*Pdisability*	38,847	0.1049	0.3064	0	1	0.0881	0.1064

## 3 Results

### 3.1 Base regression analysis

Table 2 presents the estimated effects of LTCI on labor-force participation of adult children labor-force. Column (1) excludes control variables, whereas column (2) introduces time and city fixed effects, and column (3) adds the full set of individual- and parent-level controls. In all three specifications, the interaction term, the interaction term *Post×Treat* carries a positive and statistically significant coefficient, indicating that LTCI policy implementation is associated with higher labor-force participation among adult children. Notably, the magnitude of this coefficient remains relatively stable across models, suggesting that the core finding is robust to the inclusion of various controls.

**Table 2:** Base regression analysis.

Variables	(1)	(2)	(3)
	*cwork*	*cwork*	*cwork*
*Post×Treat*	0.0942 (0.1086)	0.1815^*^ (0.1075)	0.2006^*^ (0.1159)
*Cgender*			−0.0906^*^ (0.0494)
*Cage*			0.7450^***^ (0.0432)
*Ceducation*			−0.0175^***^ (0.0038)
*Cmarry*			0.0798^***^ (0.0179)
*Chukou*			0.4267^***^ (0.0480)
*Cchildnum*			−0.2721^***^ (0.0554)
*Ctongzhu*			−0.0045 (0.0181)
*Clnin*			−0.1805^***^ (0.0495)
*Cbro*			0.0099^*^ (0.0054)
*Pgender*			−0.0331^**^ (0.0139)
*Page*			−0.0050 (0.0443)
*Page2*			0.2708^***^ (0.0386)
*Phukou*			−0.0018^***^ (0.0003)
*Peducation*			−0.3630^***^ (0.0648)
*Pmarry*			0.0422^*^ (0.0244)
*Psubjecthealh*			−0.0316 (0.0383)
*Psmoke*			−0.0084 (0.0222)
*Pdrink*			0.0651 (0.0511)
*Pchronic*			0.0473 (0.0386)
*Pyibao*			0.0053 (0.0175)
*Padl*			0.0315 (0.0605)
*_cons*	1.4979^***^ (0.0485)	2.2624^***^ (0.0576)	−7.6202^***^ (1.3812)
*lnsig2u*	−0.1656^*^ (0.0985)	−0.2105^**^ (0.0894)	−0.4884^***^ (0.0894)
*N*	38,847	38,847	37,746

Standard errors in parentheses *p < 0.1, **p < 0.05, ***p < 0.01.

The results in column (3) further show that, among child-level covariates, being male (*Cgender*) is negatively associated with labor-force participation, while factors such as age (*Cage*) and marital status (*Cmarry*) exhibit positive associations. Household registration in a non-rural region (*Chukou*) also increases the likelihood of employment. Regarding parent-level variables, parental gender (*Pgender*), age-squared (*Page2*), and marital status (*Pmarry*) have significant effects.

Overall, these findings suggest that LTCI coverage plays a key role in facilitating labor-force participation of adult children, above and beyond the influence of individual and household characteristics.

### 3.2 Robustness test

This section presents two robustness checks to validate the DID results. First, we examine whether the parallel trends assumption hold by investigating whether labor-force participation of adult children labor-force in treatment and control regions followed similar trajectories before LTCI implementation. Second, we conduct a placebo test by randomly assigning “treatment” status to cities to ensure that the observed effects are indeed attributable to LTCI.

#### 3.2.1 Parallel trend assumption

For the DID estimator to be consistent, the treatment and control groups must exhibit parallel trends in the outcome variable prior to policy implementation. In this study, parallel trends imply that labor-force participation of adult children labor-force should evolve similarly across the LTCI and non-LTCI regions before the LTCI launch, with any subsequent divergence attributed to the LTCI intervention.

**Figure 1:**
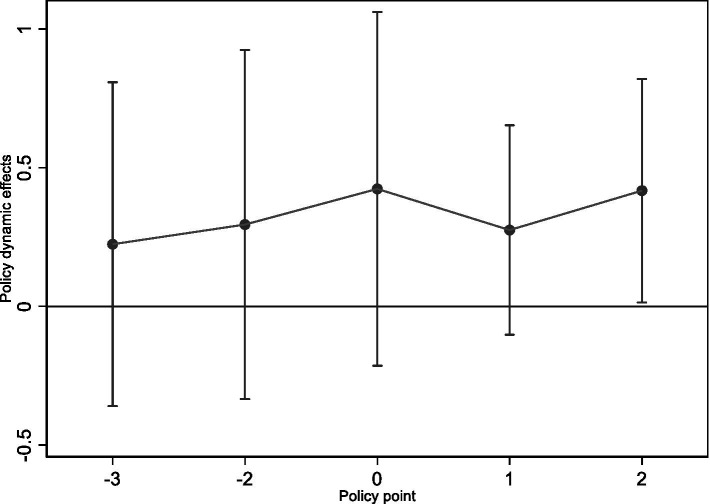
Parallel trend test results.

Because different regions initiated LTCI in different years, we apply a multi-period DID framework that aligns each individual’s time relative to their region’s specific implementation year. Specifically, we re-center the timeline such that the year before LTCI implementation serves as the baseline. Figure 1 plots the average treatment effects relative to the baseline year. The results indicate that no significant difference exists between the treatment and control groups prior to LTCI adoption, whereas a marked divergence emerges after LTCI implementation. This pattern supports the validity of the parallel trends assumption.

#### 3.2.2 Placebo testing

The core logic of the placebo test in a DID setting is to randomly assign “treatment” status to a subset of cities and estimate the effect on labor-force participation of adult children labor-force under this artificial scenario. If the estimated coefficients remain significant and similar in magnitude to the main findings, unobserved confounders rather than actual LTCI policies could be driving the results.

**Figure 2:**
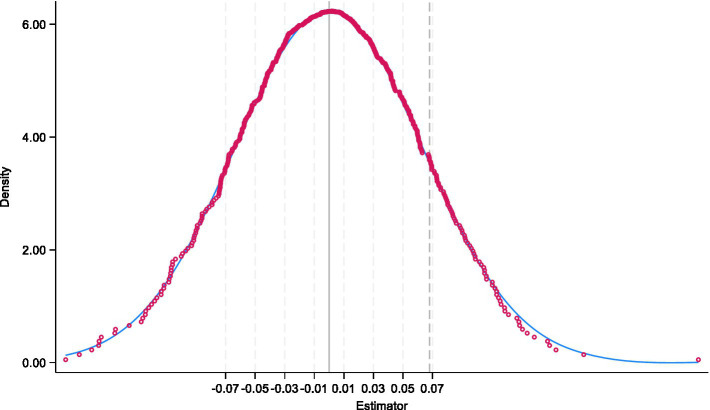
Placebo test results.

To implement this, we repeatedly draw random sets of cities as the “dummy treatment group” and re-estimate the model 500 times. Figure 2 plots the distribution of the estimated LTCI coefficients and their *p*-values. The horizontal axis shows the coefficient estimates for adult children’s labor-force participation, while the vertical axis indicates probability density. The kernel density curve centers around 0, which is substantially below the benchmark estimate from column (3) of Table 2 (indicated by the vertical dashed line). This evidence rules out the possibility that our main results are spurious or driven by latent factors. Instead, it strongly suggests that the observed increase in labor-force participation among the treatment group truly reflects the local implementation of LTCI.

### 3.3 Heterogeneity analysis

LTCI influences labor-force participation of adult children labor-force, we perform subgroup analyses by gender, age, cohabitation, and skill level. Tables 3, 4 present the corresponding regression results.

**Table 3:** Heterogeneity analysis: gender and age.

Variables	(1)	(2)	(3)	(4)
	Female	Male	Cage≥45	Cage<45
*Post×Treat*	0.1220 (0.1075)	0.3110^**^ (0.1582)	0.0771 (0.1715)	0.3719^**^ (0.1533)
*Control*	YES	YES	YES	YES
*_cons*	−8.2230^***^ (1.6296)	−6.5309^***^ (1.8240)	−4.8932^***^ (1.8205)	−1.5905 (2.8205)
*lnsig2u*	−0.3886^***^ (0.0925)	−0.7500^***^ (0.1824)	−0.6682^***^ (0.1287)	−0.5000^***^ (0.1365)
*N*	18,511	19,149	22,817	14,841

Standard errors in parentheses *p < 0.1, **p < 0.05, ***p < 0.01.

**Table 4:** Heterogeneity analysis: cohabitation and skill level.

Variables	(1)	(2)	(3)	(4)	(5)
	Not living with parents	Living with parents	Low-skill level group	Medium skill level group	High skill level group
*Post×Treat*	0.2136^*^ (0.1162)	−0.2830 (0.2271)	0.1905^*^ (0.1040)	0.4534 (0.3114)	0.1804 (0.4803)
*Control*	YES	YES	YES	YES	YES
*_cons*	−7.4919^***^ (1.3898)	−11.2050^***^ (3.9547)	−6.2545^***^ (1.3937)	−14.9400^***^ (2.9777)	−7.9280^*^ (4.5351)
*lnsig2u*	−0.4576^***^ (0.0928)	−0.7252 (0.5873)	−0.7361^***^ (0.1098)	−0.1485 (0.2042)	−1.0963^**^ (0.4303)
*N*	34,720	2,714	29,834	4,645	2,449

Standard errors in parentheses *p < 0.1, **p < 0.05, ***p < 0.01.

#### 3.3.1 Gender

Columns (1) and (2) of Table 3 examine potential gender differences in the impact of LTCI. Among men, LTCI exhibits a significant positive impact on labor-force participation ( $\alpha_{1}$ =0.3110, *p* < 0.05). By contrast, the effect for women, although positive, is not statistically significant. These results suggest that LTCI is more effective in stimulating labor supply among men than women, potentially reflecting traditional gender norms and caregiving responsibilities in China (27). Although adult children share common incentives, the link between traditional gender stereotypes can affect caregiver burden and coping strategies (28, 29). Despite the availability of formal care services through LTCI, women may still shoulder a larger share of family caregiving, limiting the impact on their labor-force participation (30, 31).

#### 3.3.2 Age

We next stratify the sample by adult children’s age, results are shown in Table 3, columns (3) and (4). Among adult children under 45, LTCI implementation significantly increases labor-force participation ( $\alpha_{1}$ =0.3719, *p* < 0.05). This result implies that younger adult children—who may be at an earlier and more flexible stage in their career—benefit more from the availability of LTCI, likely due to reduced time and financial pressures associated with parental caregiving. In contrast, for adult children aged 45 and above, the LTCI coefficient ( $\alpha_{1}$ =0.0771) is not statistically significant. One plausible explanation is that older adult children, themselves approaching retirement or balancing other familial obligations, do not experience as large a gain in labor supply from LTCI. Hence, while younger cohorts are incentivized to maintain or increase their workforce engagement, older cohorts may find it less feasible to do so.

#### 3.3.3 Cohabitation

In Table 4, columns (1) and (2) report heterogeneity by cohabitation with parents. For adult children who do not reside with their parents, the coefficient on LTCI ( $\alpha_{1}$ =0.2136, *p* < 0.10) indicates a statistically significant increase in labor-force participation following policy implementation. This finding aligns with the notion that physical distance from parents may reduce residual caregiving responsibilities, rendering formal care services especially helpful in alleviating any remaining caregiving burden.

However, for adult children who live with their parents, LTCI does not significantly affect labor-force participation. Even if formal care becomes more accessible or affordable, co-resident adult children may continue providing a larger share of caregiving tasks. Consequently, the overall effect on their employment decisions remains muted.

#### 3.3.4 Skill level

Finally, columns (3)–(5) of Table 4 explore heterogeneity by skill level, defined by educational attainment. Adult children with junior high school education or below are categorized as low-skilled, those with secondary or high school education as medium-skilled, and those with college education or above as high-skilled.

Among the low-skilled subgroup, LTCI significantly increases labor-force participation ( $\alpha_{1}$ =0.1905, *p* < 0.10). In contrast, the effects for both medium-skilled and high-skilled groups are positive but not statistically significant. These differences suggest that lower-skilled workers may be more sensitive to incremental caregiving relief provided by LTCI, possibly because they have fewer employment alternatives and face higher opportunity costs when devoting time to caregiving. By contrast, higher-skilled individuals may already be employed or have greater flexibility to balance work and caregiving, thus diminishing LTCI’s marginal impact on their labor supply.

## 4 Discussion

### 4.1 Substitution effect test

The implementation of LTCI provides new care options for older adults, particularly in light of its service payment approach in many pilot cities (14 out of 21 cities by 2018). Under this model, professional care from institutions, community workers, or specialized home services can effectively substitute for informal family-based care. To examine this substitution effect, we use the CHARLS question “Who helps you with dressing, bathing, eating, etc.?” and code assistance from employed caregivers, volunteers, nursing home personnel, or community workers as formal care (value = 1). Care offered by spouses, children, or other family members is coded as informal care (value = 0).

As shown in column (1) of Table 5, the LTCI pilot policy significantly increases the likelihood of using formal care. This finding aligns with prior evidence that subsidized LTC services can reduce reliance on informal care within the family, allowing caregivers to reallocate time to alternative activities, such as employment (2, 8, 11, 32).

**Table 5:** Mechanism testing.

Variables	(1)	(2)	(3)
	Formal	Offspring work	Parents work
*Post×Treat*	24.9720^***^ (6.5429)	0.2006^*^ (0.1159)	−0.0829 (0.1084)
*Control variables*	YES	YES	YES
*_cons*	−60.1598^**^ (28.2664)	−7.6202^***^ (1.3812)	−1.2864 (2.3494)
*lnsig2u*	1.8378^***^ (0.5557)	−0.4884^***^ (0.0894)	−0.1950^**^ (0.0865)
*N*	2,924	37,746	37,634

Standard errors in parentheses *p < 0.1, **p < 0.05, ***p < 0.01.

### 4.2 Anticipation effects test

Although the proportion of households that actually use LTCI within the sample is relatively small, the anticipated effects of LTCI can still meaningfully influence labor-force participation among adult children. Because children may adjust their current behavior (e.g., adjust labor-force participation earlier) based on expectations of future LTCI coverage. Any positive impact on their labor outcomes is more plausibly linked to the expectation that LTCI will reduce future caregiving costs or uncertainties. In other words, if adult children foresee that their caregiving burden could be lessened when their parents need care, they may be more willing to engage in or remain in the workforce.

To assess this, we compare the effect of LTCI on labor-force participation of adult children labor-force versus its effect on parents’ labor-force participation. As shown in Table 5, column (2) and (3), the LTCI coefficient is statistically insignificant for parents’ employment but positive and significant for adult children’s employment. This discrepancy indicates that LTCI exerts its strongest effect through anticipation, rather than immediate utilization. In other words, even though parents may not yet be fully utilizing LTC benefits, adult children respond to the expectation of reduced caregiving obligations, thus increasing their labor-force participation.

## 5 Conclusion

Drawing on four waves of the China Health and Retirement Longitudinal Study (2011, 2013, 2015, and 2018) and employing a DID framework, this study investigated how LTCI affects labor-force participation of adult children labor-force in Chinese pilot cities. The results point to several key findings. First, LTCI significantly increases adult children’s labor market engagement, and this effect remains robust under multiple validity and stability checks. Second, the “substitution effect” (i.e., providing professional care to ease the family caregiving burden) and the “anticipation effect” (i.e., reducing psychological uncertainty about future care) both play prominent roles in explaining LTCI’s positive impact. Third, the labor-supply response to LTCI varies by demographic subgroup, being more pronounced among men, adults under 45, and cohabitation without parents. Finally, the beneficial effect of LTCI is strongest for low-skilled workers, suggesting that educational attainment and associated labor market opportunities moderate LTCI’s influence.

Based on our findings, we propose the following recommendations: At the governmental level, we propose to provide families with higher subsidies for formal care to maximize the substitution effect by significantly reducing the out-of-pocket costs of skilled care. This will allow adult children to participate more fully in the labor market. In addition, expanding eligibility for long-term care insurance to include middle-aged families whose parents are not yet incapacitated will strengthen the anticipatory effect by building up insurance expectations early on and further increasing work incentives. At the community level, targeted outreach campaigns in neighborhoods can raise awareness of LTCI benefits, ensuring households are well-informed and can access available support. Within households, we recommend promoting caregiver training programs to enhance adult children’s ability to navigate formal care systems, reducing perceived burdens and increasing confidence in balancing work and caregiving responsibilities. Despite these contributions, the study faces certain limitations. The available data restricts the sample to four survey waves (2011–2018), and missing observations in CHARLS required reliance on an unbalanced panel, potentially affecting the precision of the estimates. As richer, more complete data on LTCI pilots and family caregiving become available, further research can delve more deeply into the mechanisms and long-term impacts of LTCI on labor supply, potentially informing policy refinements that bolster labor-force participation while ensuring quality care for older adults.

## Data Availability

Publicly available datasets were analyzed in this study. This data can be found at: data source: China Health and Retirement Longitudinal Study (CHARLS) repository Direct download link: https://charls.pku.edu.cn/.

## Footnotes

^1^Data Source: “2023 Statistical Bulletin on the Development of Medical Security,” National Healthcare Security Administration website.
